# How Should We Measure and Deal with Office Blood Pressure in 2021?

**DOI:** 10.3390/diagnostics11020235

**Published:** 2021-02-03

**Authors:** Annina S. Vischer, Thilo Burkard

**Affiliations:** 1 Medical Outpatient Department and Hypertension Clinic, ESH Hypertension Centre of Excellence, University Hospital Basel, 4031 Basel, Switzerland; thilo.burkard@usb.ch; 2 Cardiology Department, University Hospital Basel, 4031 Basel, Switzerland

**Keywords:** arterial hypertension, blood pressure measurement, guidelines, outcome studies, office blood pressure

## Abstract

Arterial hypertension is a major risk factor for cardiovascular disease worldwide. Office blood pressure measurements (OBPMs) are still recommended for diagnosis and follow-up by all major guidelines; however, the recommended procedures differ significantly. In analogy, major outcome studies usually apply OBPMs, again, with a variety of procedures. This variety of OBPM procedures complicates the comparability between studies and challenges daily clinical practice. In this narrative review, we compile the most recent recommendations for office blood pressure measurement together with the major limitations and strategies and how these could be overcome.

## 1. Introduction

Four thousand years ago, the Chinese Emperor Huang-Ti was aware of the changing characteristics of the pulse and realized that if people ate too much salt, they had hard pulses and tended to suffer strokes [1,2]. It took all the time to 1733 for Stephen Hales to undertake his famous experiments demonstrating that a horse’s blood rose to a height of 8 feet and 3 inches in a glass tube positioned in the carotid artery [1,2]. Nearly 300 years after the discovery of blood pressure measurement (BPM), arterial hypertension (AHT) is the leading preventable cause of premature death worldwide, with almost a third of the world population affected [3]. In 2019, an incredible 90% of the world population, 4.06 billion people, were found to have elevated systolic blood pressure (BP), defined as any systolic BP values over 110 to 115 mmHg. This value was used for the calculation, as below this threshold, the risk for morbidity and mortality, at least at the population level, is theoretically minimized [4]. Even more disturbing is that about a third of the population suffering from AHT is still unaware of having AHT and is therefore deprived of potentially life-saving treatment and exposed to an unnecessary risk [3]. All it would take to diagnose AHT is measuring BP appropriately.

The risk of death from strokes, ischemic heart disease and other vascular causes correlates linearly with the BP measured, starting from as little as 115/75 mmHg, as mentioned above, though there is a lack of data for even lower values [5]. Conversely, the risk reduction achieved by, for example, medical treatment or lifestyle changes correlates equally linearly with BP reduction in mmHg, at least down to 130/80 mmHg [5,6]. For example, the risk of death from strokes, ischemic heart disease or other vascular causes approximately doubles for every 20 mmHg systolic BP increase and 10 mmHg diastolic BP increase. Conversely, the risk drops by about 10% for stroke death and by about 7% for ischemic heart diseaseor other vascular death for every 2 mmHg lower systolic BP in the age group of 40–49 years [5]. On the other hand, there are data showing that by reducing BP to less than 120/70 mmHg in high-risk patients with established arterial hypertension, there may be an increase in cardiovascular outcomes such as cardiovascular death and all-cause deaths [7]. The risk is not only dependent on the absolute values, however. There is increasing evidence that a difference between both arms, especially a difference of >10 mmHg, is associated with an increased risk of all-cause mortality, cardiovascular mortality, and cardiovascular events [8] and should trigger further investigations regarding supra-aortal atherosclerosis [9,10]. Therefore, in order to assess and interpret these risks correctly for both high and low BP, we have to know what we measure when taking BP and thus need to measure BP correctly.

## 2. Guidelines’ Recommendations for Blood Pressure Measurement

**Figure 1:**
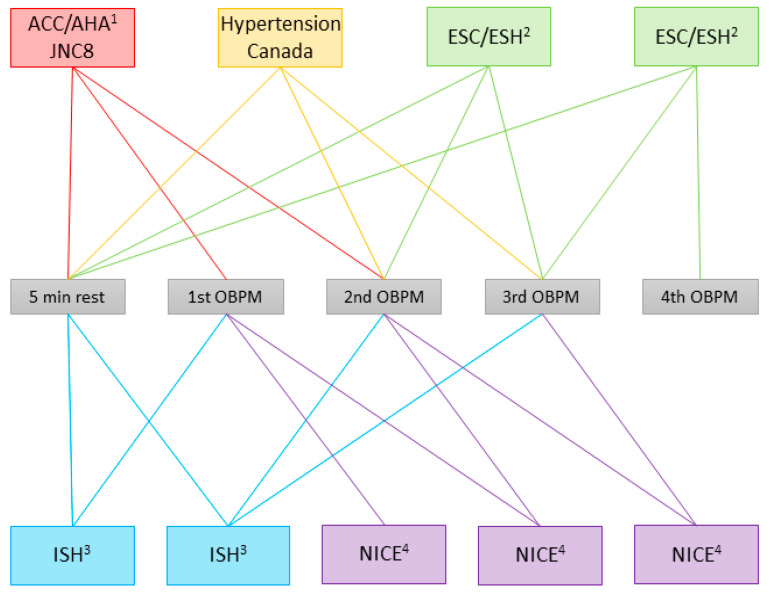
Office blood pressure measurement (OBPM) procedure recommended in the guidelines. The colored boxes enclose guidelines advocating the same procedure; the lines in the same color connect them to the measurements used to calculate the documented OBPM, and to the rest period, if applicable. Multiple boxes represent the individual guidelines if the recommendation varies depending on the situation. ACC/AHA: American College of Cardiology/American Heart Association [11]; JNC8: Eighth Joint National Committee [12]; Hypertension Canada [13]; ESC/ESH: European Society of Cardiology/European Society of Hypertension [9]; ISH: International Society of Hypertension [10]; NICE: National Institute for Health and Care Excellence [14]. ^1^ Obtain readings on ≥2 occasions. ^2^ Additional measurements should be applied only if the first two readings differ by >10 mmHg. BP is recorded as the average of the last two BP readings. ^3^ At each visit, take 3 measurements with 1 min between them. Calculate the average of the last 2 measurements. If BP of first reading is <130/85 mmHg, no further measurement is required. ^4^ If the BP is ≥140/90 mmHg, take a second measurement during the consultation; if the second measurement is substantially different from the first, take a third measurement. Record the lower of the last 2 measurements as the clinic blood pressure.

All major guidelines (the American College of Cardiology/American Heart Association (ACC/AHA), the Eighth Joint National Committee (JNC8), Hypertension Canada, the European Society of Cardiology/European Society of Hypertension (ESC/ESH), the International Society of Hypertension (ISH), and the National Institute of Health and Care Excellence (NICE)) allow using office blood pressure measurements (OBPMs) to diagnose AHT and to base treatment adaptions on [9,10,11,12,13,14]. The guidelines agree that OBPMs should be taken in a quiet room, in a sitting position, with uncrossed legs, without talking, with a validated blood pressure measurement device, and with an adequately sized cuff, without eating, smoking or exercising before the measurement. Several guidelines also recommend taking OBPMs on both arms at least once, methodologically, preferably in a simultaneous rather than a sequential manner to reduce the bias of BP fluctuations over time, and subsequently using the arm with the higher value as a reference [9,10,11,13,14]. Especially in the elderly, and patients with diabetes, they recommend screening for orthostatic hypotension by also measuring BP in a standing position [9,10,11,13,14]. Regarding whether or not a resting period should precede the OBPMs, the number of measurements to be taken and which of these measurements should be used to calculate the value documented as the final OBPM reading, the guidelines disagree, however (Figure 1). One of the selected sets of guidelines, the ACC/AHA guidelines, also recommend repeating the measurements on multiple visits before diagnosing AHT [11]. Some guidelines recommend taking a different number of measurements based either on the absolute value of the first measurement [10,14] or on the difference between the first two measurements [9,14].

Despite these differences in recommended procedures, most guidelines agree on a threshold of 140/90 mmHg for the definition of AHT, but not all. Distinctly, the ACC/AHA chose a lower threshold, in comparison to the other guidelines, of 130/80 mmHg to define AHT [11], whereas the JNC8 guidelines do not define a threshold for the diagnosis of AHT but recommend treatment initiation in patients ≥60 years with BP ≥ 150/90 mmHg, and for ≥140/90 mmHg in patients <60 years, respectively. The British NICE guidelines, on the other hand, require a combination of elevated OBPM values and elevated out-of-office BPMs such as ambulatory blood pressure measurements (ABPMs) or home blood pressure measurements (HBPMs). The threshold values are summarized in Table 1.

## 3. Basis for Recommendations

**Figure 2:**
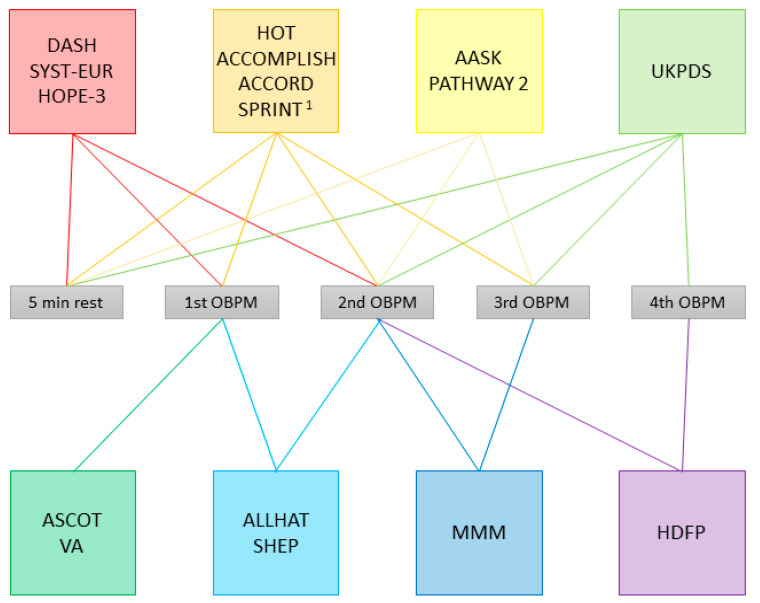
Office blood pressure measurement (OBPM) procedures applied in selected studies. The colored boxes enclose studies applying the same procedure; lines in the same color connect the box to the measurements used to calculate the documented OBPM, and to the rest period, if applicable. DASH: Dietary Approaches to Stop Hypertension [15,16]; SYST-EUR: Systolic Hypertension in Europe [17,18]; HOPE-3: Heart Outcomes Prevention Evaluation-3 [19]; HOT: Hypertension Optimal Treatment [20]; ACCOMPLISH: Avoiding Cardiovascular Events through Combination Therapy in Patients Living with Systolic Hypertension [21]; ACCORD: Action to Control Cardiovascular Risk in Diabetes [22]; SPRINT: Systolic Blood Pressure Intervention Trial [23,24]; AASK: African American Study of Kidney Disease and Hypertension [25,26]; PATHWAY 2: Prevention And Treatment of Hypertension With Algorithm-based therapy [27,28]; UKPDS: United Kingdom Prospective Diabetes Study Group [29]; ASCOT: Anglo-Scandinavian Cardiac Outcome Trial [30,31]; VA: Veterans Administration Cooperative Studies [32,33]; ALLHAT: The Antihypertensive and Lipid-Lowering Treatment to Prevent Heart Attack Trial [34]; SHEP: Systolic Hypertension in the Elderly Program [35]; MMM: May Measurement Month [36,37]; HDFP: Hypertension Detection and Follow-up Program [38]. ^1^ Procedure as per protocol, unattended in 4082/9361 subjects.

The assumed basis for the recommendation of the application of OBPMs is that most major outcome studies use OBPMs in their protocols, though none of the guidelines state which exact studies they base their specific recommendations on. One would assume that at least one protocol has been specifically linked to the prediction of an outcome such as cardiovascular events or target organ damage. However, the variety of OBPM protocols used in those outcome studies is equally large as that of the ones recommended by the guidelines. The procedures applied by a selection of major hypertension trials are depicted in Figure 2.

Whereas some studies applied a procedure that was later recommended by a set of guidelines, such as AASK or PATHWAY 2 [25,26,27,28], which used the mean of the second and third OBPMs, the method recommended by Hypertension Canada [13] and ISH, at least in some patients [10], there are several, which use similar but not quite equal methods. For example, DASH, SYST-EUR and HOPE-3 use the mean of the first two OBPMs after 5 min of rest [15,16,17,18], which is also recommended by ACC/AHA [11], albeit in a slightly modified way, as the guidelines recommend basing decisions on measurements taken on at least two different occasions. Nonetheless, there are several guidelines, especially those that recommend different procedures depending on an arbitrary cut-off value [9,10,14], for which we have not found any trial evidence from applying the same procedure.

## 4. What Are the Consequences of the Differences between the Recommended Procedures?

### 4.1. Single Versus Repeated Measurements

Disregarding all recommendations for OBPMs, many colleagues report usually performing single OBPMs [39]. Though this practice is recommended by some guidelines in the case of a normal BP [10,14], our group has shown that single compared to repeated OBPMs result in significantly different values [40]. By comparing the first single OBPM with the mean of the second to the fourth OBPMs in the same measurement session, we found that only 45% of the systolic values were within 5 mmHg in terms of differences [40]. A quarter of the systolic values differed by even more than 10 mmHg [40]. Considering the risk alteration correlating with 5 or 10 mmHg, the persistent practice of utilizing single measurements is alarming [5].

Repeated measurements, in general, but especially in the context of BPMs, follow a regression to the mean pattern [41]. This concept, first described by Francis Galton as “regression towards mediocrity”, describes the idea that non-random, thus repeated, measurements have a tendency to converge towards the population mean [41,42]. Translating this phenomenon to BP means that repetitive BPMs can be either decreasing or increasing, depending on the difference from the population mean [43]. Keeping this phenomenon in mind, it is not surprising that the mean of the second to fourth measurements is, on average, lower than the first measurement in the same OBPM session following an “individual” regression to the mean or baseline pattern [40]. Maybe less expected, but also explainable by a regression to the mean pattern (which is not unidirectional), seems a phenomenon we named short-term masked hypertension: patients with a normal first OBPM but then an elevated mean of the second to fourth OBPMs [40,43]. In our study cohort, we found this phenomenon in 3% of normotensive participants [40]. Most interestingly, we found no predictors for this phenomenon, which means that the occurrence of short-term masked hypertension can never be excluded [40]. From this effect, we can conclude that a single OBPM is not enough for properly assessing BP.

### 4.2. Difference between the Procedures Recommended by Guidelines

There is a difference not only between single and repeated measurements, but also between the different procedures recommended by guidelines stipulating repeated measurements, as our group has extensively studied.

First, we compared the Canadian to the ACC/AHA guidelines: For this comparison, we used the OBPMs of 802 participants and calculated the BPs from the same measurement set (four consecutive BP measurements under standardized conditions) once according to the ACC/AHA and once according to the Hypertension Canada guidelines [44]. Applying the procedure recommended by the ACC/AHA guidelines, though adapted to a single patient visit, resulted in a statistically non-significant increase in AHT prevalence of 4% in comparison to the procedure recommended by the Hypertension Canada guidelines, even without including the different AHT threshold definitions, but with all the BP values classified according to ACC/AHA into normal (<120/80 mmHg), elevated (120–129/<80 mmHg) or hypertensive (≥130/80 mmHg) BP [44]. Crossing the border from Canada to the United States of America leads to a reclassification of 15% of the normotensive individuals to patients with elevated or hypertensive blood pressure values, only due to the calculation procedure for the values [44].

Later, we compared the ACC/AHA to the ESC/ESH guidelines for the same cohort of participants: Again, all the BP values were classified according to ACC/AHA but calculated once according to the ACC/AHA and once according to the ESC/ESH procedure. This time, there was a statistically non-significant increase in AHT prevalence of 6% upon applying the ACC/AHA procedure instead of the ESC/ESH procedure [45]. When all the BP values were classified according to ESC/ESH instead of ACC/AHA, the increase even rose to 25% and became statistically significant [45].

**Figure 3:**
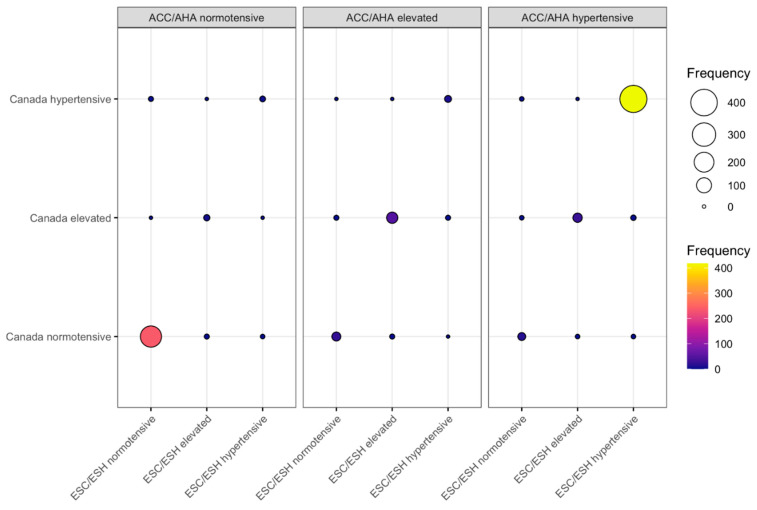
Comparison of blood pressure (BP) classifications of office blood pressure measurements (OBPMs) calculated according to the procedures recommended by the American College of Cardiology/American Heart Association (ACC/AHA) [11], European Society of Cardiology/European Society of Hypertension (ESC/ESH), [9] and Hypertension Canada [13], respectively. Data from 802 participants of the iPARR study [44,45]. Each participant had four consecutive OBPMs taken, from which the BP values were calculated according to the three sets of guidelines. All blood pressure values were categorized according to the ACC (normotensive, <120/80 mmHg; elevated, 120–129/<80 mmHg; hypertensive, ≥130/80 mmHg). The abscissa represents the values calculated according to ESC/ESH; the ordinate, the values calculated according to Hypertension Canada. The panels represent the categories decided upon through the calculation according to ACC/AHA. Both the color and size of the balloon represent the number of participants categorized in each category.

Extrapolating the data from our published papers [44,45], we can show that the ACC/AHA, the ESC/ESH and the Hypertension Canada guidelines best agree, though not completely, in the categories “normotensive” and “hypertensive” when the same OBPMs are calculated according to the recommendations of each set of guidelines and classified according to the ACC/AHA thresholds (Figure 3). Even in those two categories, there are individual patients, however, who qualify for different categories (both lower and higher) upon applying one or another OBPM procedure. Furthermore, patients who would be classified as having an “elevated” BP when applying the ACC/AHA procedure would, only in approximately 50% of cases, equally be classified as “elevated” by the other two procedures. This means that almost 50% of those patients in the “elevated” category would be classified as either “normotensive” or “hypertensive” upon applying another calculation procedure, e.g., the one recommended by ESC/ESH or Hypertension Canada. Regarding the differences between these three OBPM procedures, one has to keep in mind that the ESC/ESH and Hypertension Canada guidelines share the same procedure for about two thirds of the participants [45].

Though on a population basis, these differences may not all be statistically significant and seem minor, they may play an important role for our individual patients. Clearly, underestimating BP puts the patient in jeopardy regarding cardiovascular events [5]. On the other hand, the overestimation of BP leads to the overtreatment of the patient, which may also put the patient at risk again, considering the signs of an increased risk of cardiovascular events, at least in some studies, if the BP is lowered to values below 120/70 mmHg [7]. In addition, being labeled as hypertensive has implications not only for cardiovascular management, risk assessment and therapy, but also for psychological well-being [46,47]. One has to be aware that a patient who is labeled with a diagnosis of AHT is at an elevated risk of experiencing psychological distress [46]. There are even reports that patients labeled as hypertensive are more often absent from work than those patients not aware of their hypertension [48]. The absenteeism could not be explained by the degree of hypertension, the treatment, or the BP control reached, but was interpreted as a direct consequence of the labeling as hypertensive and consequential adoption of a sick role [47,48].

### 4.3. Adapting the Procedure Based on Arbitrary Threshold Values

Keeping the regression to the mean phenomenon in mind, it appears quite logical that the more measurements we take, the closer they should get not only to a population mean but also to the individual’s true baseline BP [41]. Therefore, it is not entirely surprising that the application of different methods, for example, using the mean of a third and a fourth measurement instead of the second and third in patients with larger differences between the first and the second measurements, leads to a comparison of apples and pears, as we demonstrated in a recent study [49]. Here, we exactly followed the ESH/ESC guidelines and calculated the BP as the mean of the second and third measurements, and as the mean of the third and fourth measurements and compared this for participants with and without a difference >10 mmHg between the first and second measurements [49]. We found that the mean of the third and fourth measurements was at least numerically lower than the mean of the second and third measurements for all the participants, with or without a difference >10 mmHg between the first two measurements [49]. Furthermore, we found that the application of the mean of the third and fourth measurements instead of the mean of the second and fourth measurements resulted in a relevant number of BP reclassifications in both directions, meaning that participants who were hypertensive according to the mean of the second and third OBPMs could be normotensive according to the mean of the third and fourth OBPMs, and vice versa [49]. On this basis, there is, to date, no scientific rationale for applying different procedures depending on arbitrary cut-off values.

### 4.4. Effect of the Rest Period

To our knowledge, the effect of the rest period as such has not been examined. However, talking, for example, leads to a BP surge of 9.1 mmHg for the systolic and 4.5 mmHg for the diastolic values [50]. The time needed for the values to return to baseline levels is about 5 min [50]. Therefore, it makes sense to have a quiet rest period before OBPMs.

### 4.5. Link from Office Blood Pressure Procedure to Outcome

To the best of our knowledge, there are no studies to date comparing the validity of different OBPM procedures as predictors for outcomes such as myocardial infarction, stroke or cardiovascular death. Only indirect comparisons are possible when looking at the risk reduction per mmHg lower BP in different individual trials—with the bias of different patient groups. It seems likely that in patients potentially eligible for an AHT diagnosis, the BP values would become lower the more often we repeated the measurements, theoretically, until we reached the population mean or, more likely, the true resting baseline BP of the patient, which we expect to reflect the risk of the patient. In reality, we would also expect a certain time factor—either the person measuring, the patient or both would become fed up with the measurements, which might lead to another increase in BP values. Therefore, a trade-off is needed to approach the “true OBPM” as much as possible without overly straining the time budget.

## 5. How to Overcome the OBPM Dilemma

### 5.1. Alternatives to Office Blood Pressure Measurements

An important way to overcome the OBPM dilemma in clinical practice is to apply possible alternatives to OBPMs, such as automated OBPMs, ABPMs and HBPMs frequently, as there is evidence that they are better predictors of target organ damage and outcomes than OBPMs [51,52,53,54,55]. These alternatives may also help to overcome common problems during OBPMs such as talking with the patient or measurements with full bladders [50,56].

#### 5.1.1. Unattended Automated Office Blood Pressure Measurements

Some oscillometric devices allow multiple automated readings performed with the patient sitting alone in the office [57]. For unattended automated office blood pressure measurements (AOBPMs), the patient should be seated in a quiet room [13]. A medical professional takes the first BPM, to verify the cuff position and validity of the measurement [13]. This first measurement should not be used for the final documentation [13]. The device then takes further readings at 1 or 2 min intervals, and the mean of these readings represents the AOBPM [13]. This method is especially adopted in Canada; accordingly, it has been endorsed and recommended by the Hypertension Canada guidelines [13]. The ESC/ESH guidelines only highlight that white coat hypertension can be reduced by this method [9].

Several studies have compared the BP results obtained through AOBPMs to other OBPM methods, to ABPMs and to HBPMs, and the results have been summarized in meta-analyses [58]. Two meta-analyses found that AOBPM correlates best with daytime ABPM [58,59]; and one, that it also correlates well with HBPM [58]. Based on these findings, the threshold for AHT is usually set at 135/85 mmHg in analogy to the AHT thresholds for awake ABPM and HBPM, which, again, correspond to the traditional 140/90 mmHg threshold for OBPM [13,59].

Unattended AOBPM has the advantage of overcoming several aspects of human error during OBPMs such as single readings, conversation with patients, and digit preference and may reduce the “white coat effect” [59,60,61].

Unattended AOBPM is criticized due to a lack of data regarding outcomes, though, for example, the SPRINT trial, a large and well-known outcome study, applied AOBPM in at least some patients [23]. In addition, some smaller studies have been published that used this method of BPM [57,62].

#### 5.1.2. Ambulatory Blood Pressure Monitoring

For ABPM, patients are fitted with an ABPM device to wear during their regular activities [2]. The device is programmed to automatically measure BP at repeated intervals, usually every 20 min during the daytime and every 30 min during the nighttime, over 24 h [2]. ABPM outperforms OBPM in the prediction of cardiovascular death, with the nighttime values and, potentially, also the 24 h mean value being the best predictors for cardiovascular events [53,54]. The main disadvantage of ABPM is the patient’s discomfort from wearing the device and its recurrent measurements, especially during the night, for which reason patients often decline this method, mainly due to fears of interrupted sleep [63]. Alternative cuff-less devices are available; however, the comparability of these devices with cuff-based ABPM remains unclear [64,65,66].

#### 5.1.3. Home Blood Pressure Measurements

For HBPM, patients measure BP themselves at home, ideally over several days, e.g., morning and evening values over 5–7 days according to defined protocols [2]. Similarly to ABPM, it has the ability to improve the prediction of cardiovascular mortality and events in comparison with OBPM [67]. Concerns that there is a relevant reporting bias regarding the values documented by the patients have, to date, not been proven [68]. However, attention has to be directed towards the devices used, as many patients use unvalidated devices, which result in inaccurate measurements [69].

### 5.2. Solution for the Application of Office Blood Pressure Measurements

Even in 2021 and the near future, there will likely be situations where OBPMs for either clinical or research purposes are inevitable. In such cases, it is recommendable to use an OBPM procedure that is likely to produce stable values close to the patient’s true BP but that is not too time consuming. In addition, it has to be easily reproducible. In our opinion, stable BP values can best be achieved by applying a rest period of five minutes and then taking OBPMs repetitively. We recommend then discarding the first OBPM, as this is usually significantly different from the subsequent measurements [40]. By using the mean of the second and the third OBPMs, good stability can be achieved [49]. Therefore, if OBPM is inevitable, we recommend using the mean of second and third OBPMs as a universal OBPM method for clinical and research purposes. Additionally, there is, in our opinion, a clear need for a consensus of all major societies to define a universal standard for attended and unattended office blood pressure measurement, which could be implemented in clinical practice and should be regarded as essential for future trials to build up a robust body of evidence.

## 6. Conclusions

In conclusion, if using OBPMs is inevitable, we recommend using the mean of second and third OBPMs after a 5 min rest period and discarding the first measurement, as the best compromise between the stability of the measurements, feasibility and reproducibility.

## Figures and Tables

**Table 1:** Blood pressure thresholds recommended for the definition of arterial hypertension (AHT) in different guidelines.

Guidelines	AHT Threshold	Reference
ACC/AHA	130/80 mmHg ^1^	[11]
JNC8	150/90 mmHg/140/90 mmHg ^2^	[12]
Hypertension Canada	140/90 mmHg ^3^	[13]
ESC/ESH	140/90 mmHg	[9]
ISH	140/90 mmHg	[10]
NICE	140/90 mmHg ^4^	[14]

ACC/AHA: American College of Cardiology/American Heart Association [11]; JNC8: Eighth Joint National Committee [12]; Hypertension Canada [13]; ESC/ESH: European Society of Cardiology/European Society of Hypertension [9]; ISH: International Society of Hypertension [10]; NICE: National Institute for Health and Care Excellence [14]. ^1^ Obtained on ≥2 occasions; ^2^ Treatment initiation for general population aged ≥ 60 years and <60 years, respectively, AHT threshold not defined; ^3^ If the OBPM is 140–179/90–109 mmHg, out-of-office BP measurements should be performed; ^4^ In combination with an ambulatory blood pressure measurement (ABPM) daytime average of 135/85 mmHg or higher, a home blood pressure measurement (HBPM) average of 135/85 mmHg or higher.

## Data Availability

Data sharing not applicable.
